# Prevalence and factors associated with erectile dysfunction in hemodialysis patients: cross-sectional study

**DOI:** 10.1192/j.eurpsy.2025.2292

**Published:** 2025-08-26

**Authors:** A. Ben Haouala, A. Balti, I. Betbout, M. Ben Mbarek, B. Amamou, A. Mhalla

**Affiliations:** 1 Psychiatry, University of Monastir, Monastir, Tunisia

## Abstract

**Introduction:**

Hemodialysis, although crucial for maintaining the survival of patients suffering from end-stage renal disease, can have severe repercussions on patients’ overall quality of life and sexuality.

**Objectives:**

To determine the prevalence and factors associated with sexual dysfunction in hemodialysis patients.

**Methods:**

This is a multicenter, descriptive, cross-sectional study with analytical aims, conducted at 3 hemodialysis units (the Mahmoud El Matri Hospital in Ariana, the El Manzah private hemodialysis center and the El Omrane polyclinic) over a 5-month period (August-December 2023). Patients’ sexual dysfunction was assessed using the IIEF5 scale.

**Results:**

Our study population comprised 73 patients. Patient ages ranged from 25 to 86 years, with a median age of 56.87 years. Twenty-eight subjects (38.4%) were between 56 and 70 years of age. Sixty patients (82.2%) were married, with 68 living with relatives. Regarding anamnestic data, organic comorbidity was found in all patients with diabetes in 67.1% and arterial hypertension in 50.7% of cases. The median duration of hemodialysis was 48 months. The frequency of hemodialysis sessions was 3 per week in 69 patients (94.5%).

Erectile dysfunction was diagnosed in 64 patients, corresponding to a prevalence of 87.7%. The dysfunction was mild in 24.7% of cases, moderate in 45.2% and severe in 17.8%.

Erectile dysfunction was significantly correlated with age over 55 with an adjusted OR = 2.43 and the presence of diabetes with an adjusted OR = 1.5.

**Conclusions:**

The prevalence of erectile dysfunction is high in hemodialysis patients. A systematic sexological approach is needed to ensure early and appropriate management.

**Disclosure of Interest:**

None Declared

